# Experimental Protein Molecular Dynamics: Broadband Dielectric Spectroscopy coupled with nanoconfinement

**DOI:** 10.1038/s41598-019-54562-8

**Published:** 2019-11-29

**Authors:** Laëtitia Bourgeat, Anatoli Serghei, Claire Lesieur

**Affiliations:** 1 AMPERE, CNRS, Univ. Lyon, 69622 Lyon, France; 2 IMP, CNRS, Univ. Lyon, 69622 Lyon, France; 3 Institut Rhônalpin des systèmes complexes, IXXI-ENS-Lyon, 69007 Lyon, France

**Keywords:** Nanoscale biophysics, Atomic and molecular physics

## Abstract

Protein dynamics covers multiple spatiotemporal scale processes, among which slow motions, not much understood even though they are underlying protein folding and protein functions. Protein slow motions are associated with structural heterogeneity, short-lived and poorly populated conformations, hard to detect individually. In addition, they involve collective motions of many atoms, not easily tracked by simulation and experimental devices. Here we propose a biophysical approach, coupling geometrical nanoconfinement and broadband dielectric spectroscopy (BDS), which distinguishes protein conformations by their respective molecular dynamics. In particular, protein-unfolding intermediates, usually poorly populated in macroscopic solutions are detected. The protein dynamics is observed under unusual conditions (sample nanoconfinement and dehydration) highlighting the robustness of protein structure and protein dynamics to a variety of conditions consistent with protein sustainability. The protein dielectric signals evolve with the temperature of thermal treatments indicating sensitivity to atomic and molecular interaction changes triggered by the protein thermal unfolding. As dipole fluctuations depend on both collective large-scale motions and local motions, the approach offers a prospect to track in-depth unfolding events.

## Introduction

Proteins produce biological activities in living organisms thanks to their 3D-structure and the associated dynamics. The 3D structure relies on chemical interactions between atoms of the amino acids that compose a protein. The molecular dynamics is based on molecular fluctuations, which produce motions from a local scale (amino acid side chain fluctuations) to a larger scale (structural relaxation) to respond to the protein folding and the protein function^1–3^. Accordingly, proteins dynamics covers several orders of magnitude of spatiotemporal motions from femtosecond to second for molecular fluctuations from Angströms to nanometers. Basically, fast motions (femtosecond to nanosecond) concern local atomic motions (vibration, side chain motions) while slow motions concern the collective motions of many atoms within larger size areas such as secondary and tertiary structural elements (microsecond to millisecond) up to domains (millisecond to second)^3^.

To track protein dynamics, several experimental and theoretical approaches are required. Ultra resolution X-ray crystallography and x-ray laser have been successfully applied on few cases to monitor time-resolution of conformational motions^4–7^. Ultra fast NMR, femtosecond simulated Raman spectroscopy and ultra fast transient Infra Red spectroscopy are measuring atomic motions from femtosecond to nanosecond^8–13^. Slower motions above nanosecond are monitored by low spatial resolution techniques such as AFM and fluorescence spectroscopy (FRET) where the molecular dynamics are inaccessible^14–16^.

Theoretical approaches such as molecular dynamics (MD) simulations have atomic resolution and cover motions up to millisecond now thanks to ANTON super computer^1^. But MD simulation at high resolution is limited in terms of size and it remains hard to assess motions above microsecond range^17,18^. Network models are also useful to study protein dynamics^19–22^.

Most successful approaches are integrative, combining experimental and multiple theoretical approaches in order to cover more length and time scale motions of protein structure dynamics^23–25^. They are particularly successful in monitoring the slow motions involved in protein assembly and pore-formation^26–32^.

Measuring the spatiotemporal dynamics of slow collective motions in proteins is crucial because it is the range where protein folding and biological activity take place. It is also the range to assess the health state of a protein as pathological mutations are distinguished from robust mutations by different collective motions^33^. Protein robustness to mutations or to external perturbations such as environmental changes relies on the diversity of structures adopted by a protein in order to function^34–37^. But there are little measurements of such structural diversity of a protein or of its sequence variants on which to explore the set of atomic and molecular interactions that sustain robustness and from which to infer potential structural and dynamics fragility^24,38,39^. This is because slow collective motions associate with short-lived poorly populated conformations or conformations globally close requiring high sensitivity and probing of atomic interaction fluctuations^40^.

To overcome these difficulties, broadband dielectric spectroscopy (BDS) was used to monitor dipole fluctuations in the frequency range from 1 Hz to 10^6^ Hz relevant to slow motions and combined with nanoconfinement for lowering the protein dynamics and enriching homogeneous conformations. The nanoconfinement was initially developed to analyze attograms (1 attogram = 10^−18^ g) and zeptograms (1 zeptogram = 10^−21^ g) of matter and investigate the dielectric behavior of matter on a length-scale becoming already comparable with molecular dimensions^41,42^. The characteristic volume of 1 attogram (considering a density of ~1 g/cm^3^) is ~10 × 10 × 10 nm, approaching thus the characteristic dimensions of one protein and offering the possibility to measure protein dynamics at the scale of the protein instead of the scale of a population of proteins.

Nanoconfined cholera toxin B subunit pentamers (CtxB_5_) were incubated at different temperatures from 60 °C to 180 °C and the dielectric signal measured after each thermal treatment. The toxin dielectric loss ε” varies with the thermal treatment showing the technic sensitivity to atomic and molecular interaction changes induced by the toxin thermal unfolding. To the best of our knowledge, investigating protein dynamics using nanoconfinement and BDS on the scale of attograms has never been performed before.

## Material and Methods

### Materials

Lyophilized cholera toxin B pentamer purchased from Sigma Aldrich (C9903) was diluted in phosphate buffered saline (PBS; 10 mM sodium phosphate, 150 mM sodium chloride, pH 7.4) at a final concentration of 2,6 mg/ml. This bulk toxin preparation was aliquoted and kept at −20 °C, thawed and frozen twice maximum, when used for the dielectric measurements. Ten-time concentrated solution of PBS solutions were purchased from BIOSOLVE and diluted 10-times in distilled water to prepare the PBS used to prepare the bulk toxin solution. In addition, the PBS solution was filtered in 22 μm filter to limit impurity contamination. The Aluminium Oxyde (AAO) Films on Al are purchased from InRedox. The AAO membrane dimensions are 10 mm * 10 mm, and they contain nanopores of 40 nm-diameters and 10-µm lengths, with 12% porosity. The AAO membrane containing the nanopores is referred to as the nanomembrane. Before use, the nanomembranes were plasma treated for twenty minutes to remove organic impurities.

### Methods

Protein sample preparation- For the dielectric measurement, a protein sample at 0,025 mg/ml was used. This protein sample was prepared by diluting 2 μl of the bulk toxin solution at 2,6 mg/ml in 204 μl of deionized water (Final pH 6,9). Since the bulk protein is prepared in PBS, the sample for the dielectric measurement contains protein, deionized water and PBS. A 200 μl drop of sample is deposited on the nanomembrane using the drop technique, then heated at 50 °C (323 K) for 15 min to evaporate the bulk water and allow proteins to enter the pores. The sample is cooled down to 30 °C (303 K) for 5 minutes with a speed of 2 K/min, placed between two Al electrodes of 7 mm and 40 mm diameter, respectively and in the cell holder for measurement. The procedure is the same for the control samples.

### Dielectric measurements

The dielectric measurements were performed on a broadband dielectric spectrometer Novocontrol Alpha analyzer over a frequency range from 1 Hz to 10^6^ Hz and over a temperature range from −80 °C (193 K) to 180 °C (453 K). The temperature ramps were carried-out with a rate of 2 K/min and a voltage of 0.2 V was applied. The dielectric loss ε”, the imaginary part of the complex dielectric permittivity, was measured as a function of temperature at different frequencies. For the temperature control, a flow of pure nitrogen gas was used in a closed cryostat, providing water-free and oxygen-free experimental conditions. The sample was first heated to 60 °C (333 K) for three hours, then cooled down to −80 °C (193 K) (Cooling, C) and maintained for 30 min before being heated back to 60 °C (Heating, H). A cycle is composed of the cooling (C) and the heating (H) temperature ramps. The sample was heated again for three hours at 60 °C and a second cycle of cooling/heating, repeated to measure the reproducibility of the signal. The curves of the cooling and heating signals of the cycle 1 and of the cycle 2 are almost superimposed for the protein sample (Supplementary Fig. S1A). This demonstrates that the proteins under study are stabilized in a state where no bulk water is present anymore and where the amount of adsorbed water is stabilized as well. It also indicates that three hours incubation at 60 °C is enough for the protein to reach a stable conformational state and this incubation time can be used for thermal treatments at higher temperatures. In contrast, the cooling signal of the PBS buffer control is higher than the heating signal showing that evaporation of the bulk water still occurs in the buffer sample after the first three hours at 60 °C (Supplementary Fig. S1B). Since this is not the case for the protein sample, it suggests more adsorbed water in the protein sample. At 1 Hz, the same is observed (Supplementary Fig. S1C and D).

From 60 °C to 180 °C, little hysteresis is observed indicating that the dielectric loss ε” does not detect different cooling and heating processes (Supplementary Fig. S2A). The protein remains in a steady state across the measurement and undergoes thermal unfolding mainly during the three hours incubation time.

The thermal denaturation of the toxin is studied using temperature ramps with a series of 3-hours thermal treatments at 100 °C (373 K), 140 °C (413 K) and 180 °C (453 K) followed by cooling/heating cycles after each treatment.

It is worth noting that one full dielectric experiment uses as little as ~5 μg of toxin.

### Dielectric data

The temperature dependence of the relaxation processes is modeled by an Arrhenius equation expressed as (Eq. 1)^43,44^.

1 $${\rm{\tau }}={{\rm{\tau }}}_{0}\,\exp \,({{\rm{E}}}_{{\rm{A}}}/{{\rm{k}}}_{{\rm{B}}}{\rm{T}})$$

where τ is the relaxation time, τ_0_ the relaxation time at high temperatures, T the temperature, E_A_ the activation energy and k_B_ the Boltzmann constant^45^.

### Control experiments

The dielectric response of the empty nanomembrane was measured for 10 min at 60 °C and from 60 °C to −80 °C to control that no dielectric dispersions are detected. The dielectric response of nanopores filled with a 200 μl drop of deionized water was measured for 10 min at 60 °C and from 60 °C to −80 °C to control that the deionized water used to prepare the sample show no dielectric dispersions too. Finally, the dielectric response of nanopores filled with a 200 μl drop of PBS buffer control, is measured following a complete temperature ramp experiment as for the protein sample. The PBS buffer control is prepared with 2 μl of PBS diluted in 204 μl of deionized water (no protein is added). This concentration is equivalent to the concentration used to prepare and measure the protein sample. This control experiment was carried out to investigate the dielectric response of PBS mixed with deionized water in the same conditions as the protein.

To make sure the toxin is in a native state when a dielectric experiment is performed, the toxin pentameric state is checked by Trp-fluorescence beforehand^46^. The sample cannot be recovered from the nanopores after the BDS experiment so it is not possible to control the state of the toxin after.

## Results

The goal of the study is to determine if combining nanoconfinement and broadband dielectric spectroscopy (BDS) allows distinguishing protein conformations by measuring different molecular dynamics. To test such a possibility, the cholera toxin B pentamer (CtxB_5_) is chosen because of its high stability and functional resistance to freeze-drying, which shows that the dehydration of the toxin does not lead to irreversible conformational changes. This is important since the dielectric measurements are performed under dehydrated conditions where the toxin sample contains PBS and only adsorbed water (Methods and Supplementary Fig. S1).

**Figure 1:**
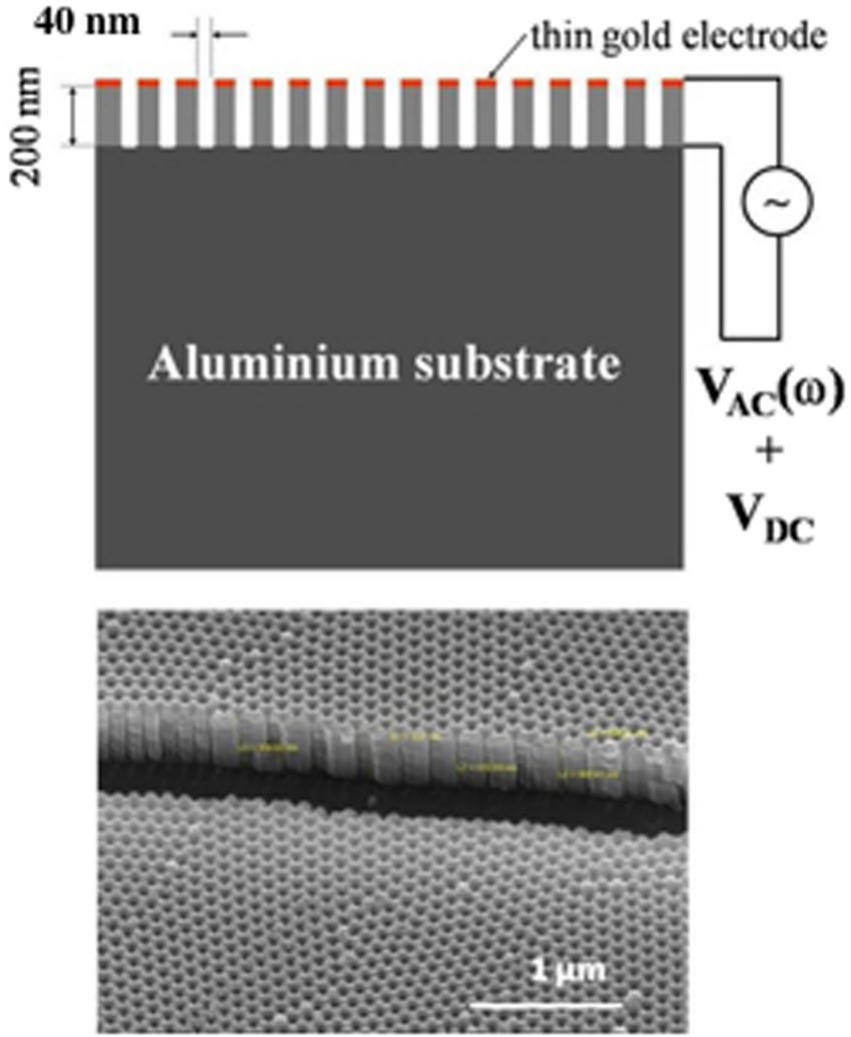
Nanopores as experimental cells to hold and measure attograms of material. The nanopores are identical, independent, and additive. Upper panel. Schematic representation of the sample cell. The lower electrode and the upper electrode are connected to the dielectric spectrometer and used to measure -in dependence on frequency and temperature- the permittivity of the sample cell. Lower panel. SEM image of the nanocontainers in cross section.

**Figure 2:**
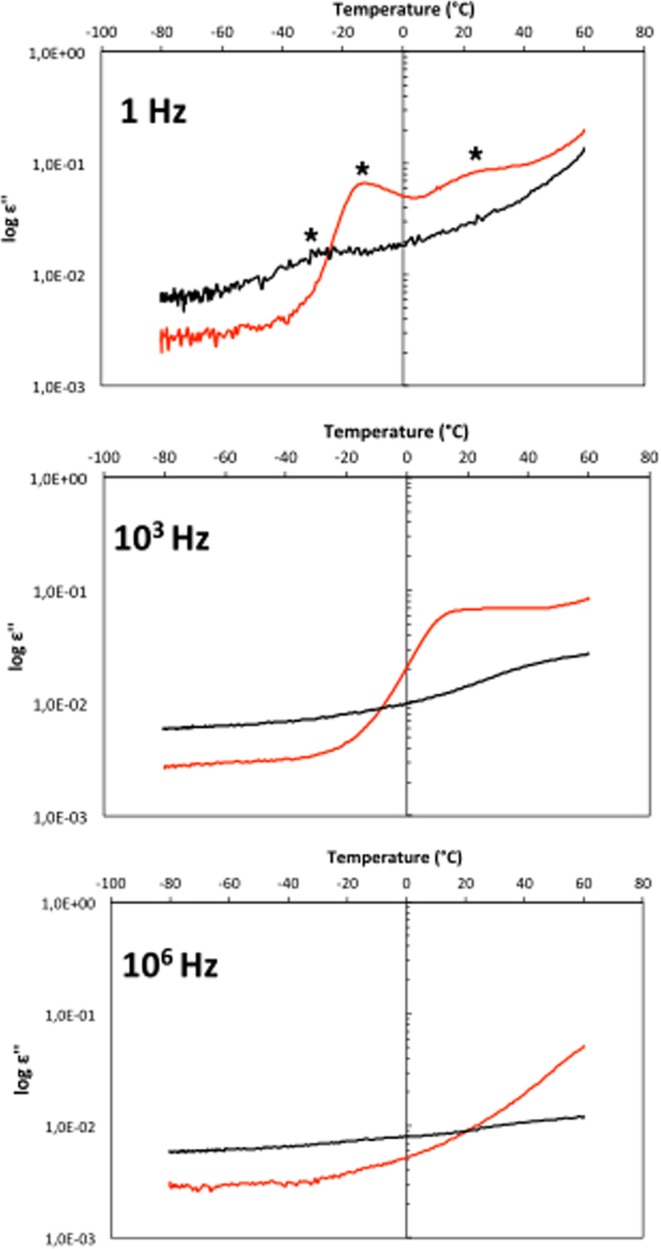
Dielectric loss as a function of temperature, at different frequencies (as indicated) for Cholera toxin B pentamer (red) and PBS buffer control (black), after a thermal treatment of 3-hours at 60 °C.

Toxin and PBS buffer control samples are deposited in the nanomembrane and the dielectric loss *ε*” of the sample is measured by BDS, the whole setup is shown on Fig. 1. The samples are incubated for three hours at 60 °C and the dielectric loss *ε*” is measured as a function of temperature (Fig. 2) (Methods). The protein and the PBS buffer control spectra can be described by several relaxation processes, which give rise to peaks in the spectra of the dielectric loss (Fig. 2, *).

At 1 Hz (Fig. 2, top panel), the protein sample presents two dipole relaxation processes and hence two molecular dynamics (MDs). The main one, narrow, is the fastest with a maximum temperature position at −14 °C (MD_1_). The second, broader and less distinct, is slower with a maximum temperature position at 30 °C (MD_2_). The broadness of MD_2_ suggests a relaxation process associated with a heterogeneous population of toxin conformations while the narrower MD_1_ peak suggests less heterogeneous toxin conformations. The buffer control also presents a relaxation process and a molecular dynamic (MD_b_) with a maximum dielectric loss *ε*” at −27 °C, so a fastest process than those observed for the toxin. At 10^3^ Hz (Fig. 2, middle panel), a relaxation process is still detectable for the protein with a maximum dielectric loss *ε*” at 13 °C (MD_1_). For the PBS buffer control, a relaxation process is also observed but not well enough to have an accurate maximum temperature position (Fig. 2, middle panel). The maximum temperature positions shift to higher temperatures with increasing frequencies. At 10^6^ Hz, neither the protein nor the PBS samples have a detectable signal in the experimental window of our investigations (Fig. 2, bottom panel).

To test whether the dielectric losses probe protein conformation and the relaxation processes two different toxin conformations, the toxin is submitted to thermal denaturation (Methods). Globular proteins go from well-packed structures with numerous atomic and molecular interactions that hold their native state at low temperatures to less compact unfolded structures at high temperatures, where conformations loosing atomic and molecular interactions open to solvents and conserved only residual structures^47,48^. Thermal stability significantly varies for proteins from mesophilic to thermophilic organisms^47,49^. The CtxB_5_ thermal stability has been studied using differential scanning calorimetry and it exhibits a single transition centered at 74 °C^50,51^. The thermal treatments disturb the toxin native atomic and molecular interactions and are hence expected to alter dipole and/or dipole environments, and consequently modify the global dielectric losses.

**Figure 3:**
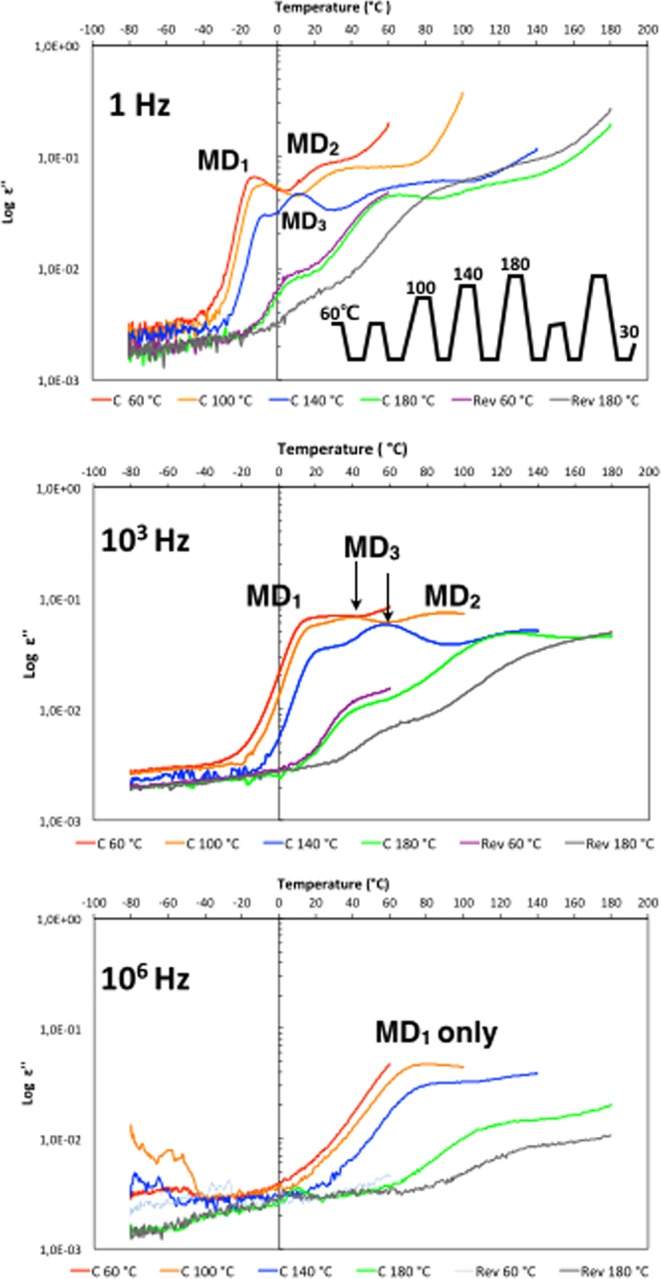
Dielectric loss as a function of temperature for Cholera toxin B pentamer, at indicated frequencies and after thermal treatments of 3-hours at 60 °C, 100 °C, 140 °C and 180 °C. Those are captioned as C 60 °C, C 100 °C, C 140 °C and C 180 °C, C for cooling, because the cooling signal is shown. After the three hours incubation at 180 °C and cooling to −80 °C, the sample is warmed back up to 60 °C for 3-hours and the subsequent cooling signal is shown, captioned Rev 60 °C for reversibility. Then the sample is warmed for 3-hours at 180 °C, and the subsequent cooling signal is shown, captioned Rev 180 °C.

Three hours thermal treatments at 100 °C, 140 °C and 180 °C are performed since it is enough time for the protein to reach a stable state at 60 °C (Methods and Supplementary Fig. S1A). After the incubation step, the dielectric loss *ε*” is measured from the incubation temperature (100 °C, 140 °C or 180 °C) to −80 °C (Cooling) and from −80 °C back to the incubation temperature (Heating) (Fig. 3). The dielectric loss *ε*” is shown as a function of temperature for the cooling process but both the cooling and heating signals are similar over the four temperatures of the thermal treatments (Methods and Supplementary Fig. S2A).

The thermal treatments leads to a loss of the relaxation process of the PBS buffer control and only conductivity is observed after treatment at 100 °C and 1 Hz frequency (Supplementary Fig. S2B). In contrast, the toxin maintains multiple relaxation processes evolving across the thermal treatments up to 180 °C and over the frequencies from 1 Hz to 10^6^ Hz, confirming that the dielectric loss of the protein is conformation specific and sensitive to atomic and molecular interaction changes (Fig. 3).

At 1 Hz, the dielectric losses are similar after the thermal treatments at 60 °C and at 100 °C, suggesting that the detected molecular dynamics and protein conformations are stable up to 100 °C. The maximum temperature positions of MD_1_ shift only from −14 °C to −7 °C and from 30 °C to 40 °C for the MD_2_. In the same time, the MD_2_ peak becomes even broader.

After incubation at 140 °C and at 180 °C, the dielectric losses change significantly more. The intensity of the dielectric loss of MD_1_ drops at 140 °C and even more at 180 °C with, in addition to a broadening of the peak; a shift of the maximum temperature positions from −7 °C to 10 °C at 180 °C. The drop of the dielectric loss intensity of MD_1_ suggests a disappearance of the average conformations associated with MD_1_ as the temperature rises above 100 °C.

The MD_2_ peak broadens and the maximum temperature position shifts from 40 °C to 80 °C after the 140 °C treatment (Fig. 3, top panel, blue curve). In contrast to MD_1_, the intensity of the dielectric loss *ε*” of MD_2_ only slightly drops when the protein is heated up to 140 °C but the dielectric loss *ε*” is undetectable after the treatment at 180 °C. This suggests as for MD_1_ that the toxin conformations associated with MD_2_ disappear with increasing temperature above 140 °C.

At 140 °C, simultaneously to the drop of the MD_1_ signal, a new MD (MD_3_) appears with a narrow peak and a maximum temperature position at 10 °C (Fig. 3, upper panel). The MD_3_ peak broadens after treatment at 180 °C and the maximum temperature position shifts to 70 °C.

MD_1_ signal drops with increasing temperatures but with no evident increase of the signals of MD_2_ and MD_3_ compensating for the loss of the MD_1_ signal. This indicates that the protein unfolds into conformations, others than MD_2_ and MD_3_, and not detected at frequencies between 1 Hz to 10^6^ Hz. This would suggest that unfolded states of proteins where most ‘native’ atomic and molecular interactions and initial dipoles are lost, might need to be investigated at lower frequencies to be detected.

To assess whether the protein initial conformation associated with MD_1_ could be recovered after the thermal treatment at 180 °C and the cooling down to −80 °C, the sample was heated back to 60 °C and kept at 60 °C for three hours (Fig. 3, purple curve). The dielectric losses measured after the treatment at 180 °C and after the subsequent three hours at 60 °C were similar, indicating that the thermal damages are irreversible and the protein does not refold into the initial conformation despite three hours at 60 °C (Fig. 3, green, purple and red curves, respectively). Finally, the sample was heated back to 180 °C and kept for three more hours and the dielectric loss measured again (Fig. 3, grey curve). The signal appears lower than after the first treatment at 180 °C and MD_1_ is almost undetectable indicating that the associated averaged conformations is almost gone. After the second treatment at 180 °C, the MD_2_ and MD_3_ peaks seem merged.

At 10^3^ Hz, MD_2_ is barely detected while the MD_1_ temperature-dependent features change similarly to 1 Hz, and the MD_3_ can already be detected at 100 °C (Fig. 3, middle panel). At 10^6^ Hz, only MD_1_ is detectable, with a drop of the dielectric loss, a shift in the maximum temperature position with the increase of the temperature of the thermal treatments as observed for lower frequencies (Fig. 3, bottom panel). The signal is noisier at temperatures below −20 °C but at 10^6^ Hz, it becomes noisier almost over the whole temperature range.

**Figure 4:**
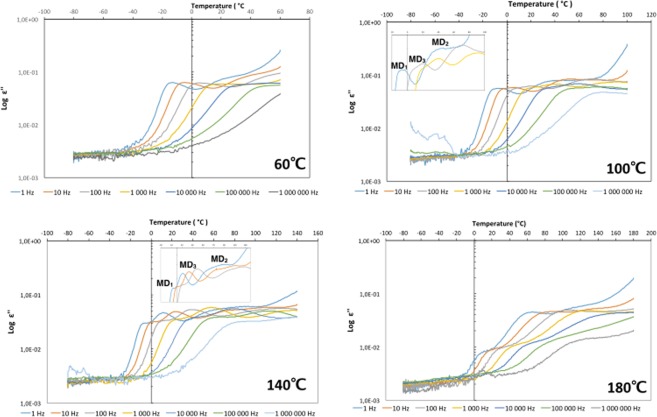
Dielectric loss as a function of temperature for Cholera toxin B pentamer, at frequencies from 1 Hz to 10^6^ Hz after three hours thermal treatments at 60 °C (Top left), 100 °C (Top right), 140 °C (Bottom left) and 180 °C (bottom right). Only few frequencies are shown in the insets for the sake of clarity.

The frequency dependency of the three relaxation processes was investigated as function of temperature (Fig. 4). MD_1_ covers both low and high frequencies from 1 Hz to 10^6^ Hz over the thermal treatments from 60 °C to 180 °C. The MD_2_ is detected only at low frequencies from 1 to 10^3^ Hz over the thermal treatments from 60 °C to 140 °C. MD_3_ covers high frequencies after the thermal treatment at 100 °C, both high and low frequencies after the thermal treatment at 140 °C, and only low frequencies after the thermal treatment at 180 °C.

**Figure 5:**
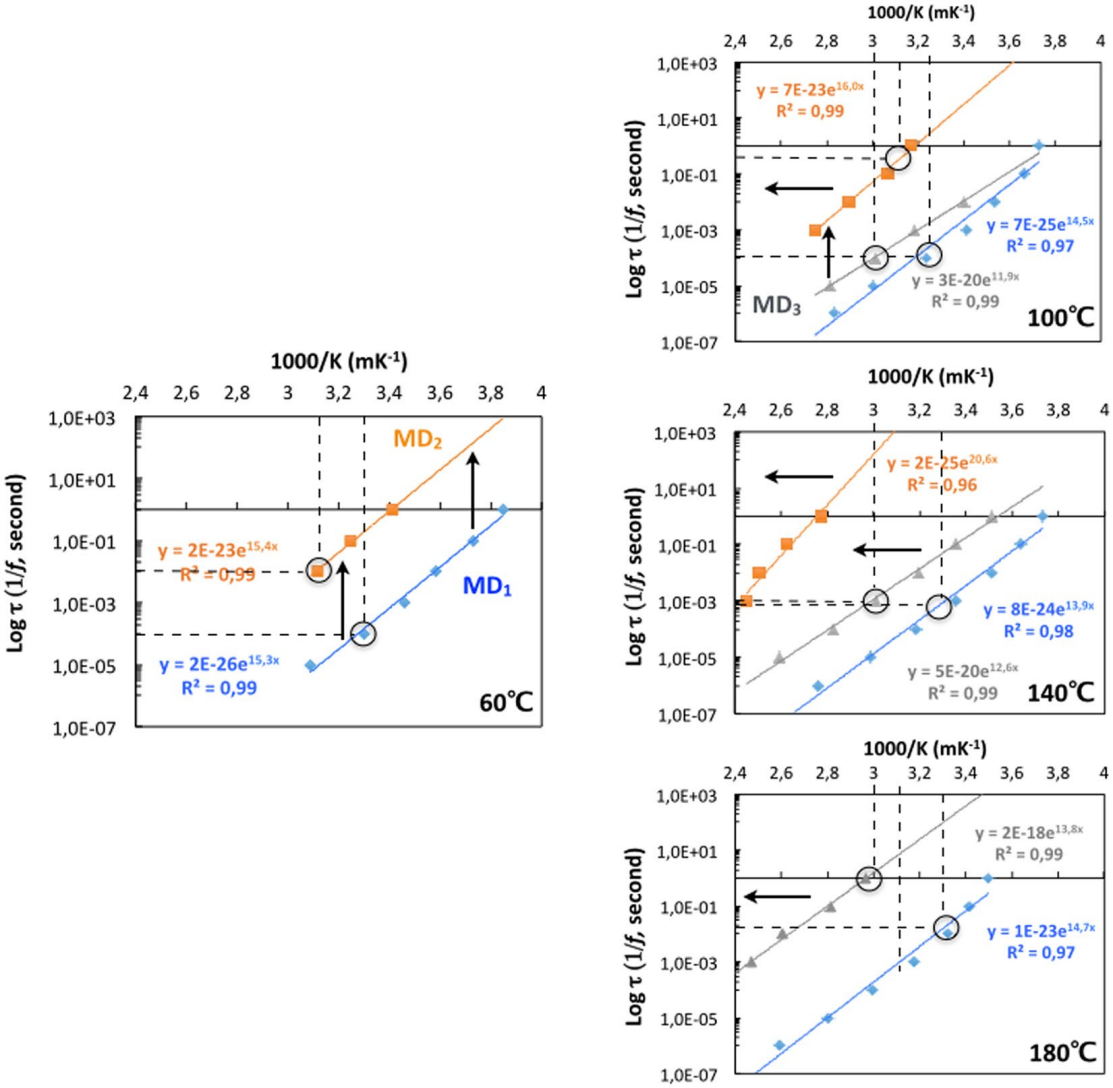
Temperature dependences of the relaxation times for the three relaxation processes identified for the Cholera toxin B pentamer after thermal treatments at 60 °C (Top left), 100 °C (Top right), 140 °C (Bottom left) and 180 °C (bottom right). Dipoles changing frequencies across relaxation processes and with the increase of the temperature of the thermal treatments are highlighted with arrows and circle, respectively.

The temperature dependencies of the relaxation times tau τ (τ* = *1/*f*) of the three relaxation processes are plotted on Fig. 5. The relaxation times linearly depend on the maximum temperature positions indicating that dipoles fluctuating at different frequencies are independent of one another and that is true for the three relaxation processes. Three signal features change with the temperature of the thermal treatments and with the relaxation processes.

First, the relaxation times of dipoles with similar maximum temperature positions become significantly slower in MD_3_ and in MD_2_ as the temperatures of the thermal treatment increase (Fig. 5, circles). In contrast, they remain similar up to 140 °C in MD_1_ and become slower only after the treatment at 180 °C (Fig. 5, circles). The relaxation times of dipoles with similar maximum temperature positions also become slower across the MDs (Fig. 5, vertical arrows). This means thermal unfolding leads to motions at larger spatial scale with no impact on the dipole local environment (no change in the maximum temperature positions). It could be interface motions or beta sheet motion for example. Moreover, regardless the maximum temperature positions, the relaxation times of MD_3_ are ten times slower at 140 °C than at 100 °C (Fig. 5, circles). Likewise, the relaxation times in MD_3_ are ten times slower than in MD_1_ at 100 °C and hundred times slower at 140 °C, regardless the maximum temperature positions. The relaxation times of dipoles in MD_2_ are thousand times slower than in MD_1_ regardless the maximum temperature positions. This indicates that dipoles fluctuating at different frequencies are independent of one another even as toxin thermal unfolding occurs. Dipole fluctuation influences as the toxin unfolds might happen during the incubation time and thus not be detected on steady state conformations.

Second, the maximum temperature positions of dipoles with similar relaxation times increase (1000/K decrease) with the increase of the temperature of the thermal treatments (Fig. 5, horizontal arrows). This is also observed across relaxation processes as the maximum temperature positions of dipoles with similar relaxation times increase from MD_1_ to MD_3_ to MD_2_. This means thermal unfolding also leads to changes in the vicinity of the dipoles (changes in the maximum temperature positions) with no impact on the dipole relaxation time (no change in tau). This could be a dipole going from a buried position to a surface position but keeping same fluctuation.

Third, dipoles change in terms of both relaxation times and maximum temperature positions. For example, there are no dipoles with slow relaxations time in MD_3_ at 100 °C but there are some at 140 °C (Fig. 5). This means the MD_3_ dipoles with fast relaxation times at 100 °C have slowed down and changed their maximum temperature positions at 140 °C. Here the thermal unfolding leads to local changes and larger scale collective motions.

The relaxation time temperature dependence of the three relaxation processes fits well with an Arrhenius equation (Methods) over the four thermal treatments at 60 °C, 100 °C, 140 °C and 180 °C (Fig. 5). They present similar activation energies of ≈27–30 kcalmol^−1^ across the three MD and for the different temperatures of the thermal treatments, except for the MD_2_ at 140 °C whose activation energy is ≈ 41 kcalmol^−1^ (Fig. 5, slopes). Such similar activation energies across the MD and the temperatures of the thermal treatments are consistent with having similar probes monitored in all processes but in different molecular environment as the toxin unfolds locally (around the dipole) or at larger scale.

## Discussion

The main narrow peak MD_1_ observed at 60 °C suggests that the nanopores work as a conformational funnel, and reduce the structural heterogeneity of the toxin in solution by constraining the 3D space where the toxin can move (nanopore diameter). We are currently testing other nanopore dimensions to investigate this hypothesis. In addition to detecting MD_1_, the nanoconfinement combined with BDS also distinguish, via their respective molecular dynamics (MD_2_, and MD_3_) two other average toxin conformations induced by the thermal treatments.

The toxin conformations and dynamics measured by BDS combined with nanoconfinement are clearly different from the toxin dynamics measured under macroscopic and hydrated conditions as illustrated by the toxin resistance to thermal treatments up to 180 °C instead of 80 °C^51^. Such impact on the toxin stability is consistent with the detection of disassembled and/or unfolding species, rarely observed otherwise, made more stable under the conditions. The nanoconfinement, the dehydration or a combination of both could be responsible for the high temperature-resistance. Dehydration conditions are known to impact protein dynamics^52–54^. Hydrogen bonding might be reduced under dehydration while the presence of the PBS buffer could favor salt bridges and lead altogether to a more stable toxin. This would be consistent with the role of stabilizing salt bridges on temperature resistance observed in thermophilic proteins^55–57^. BDS experiments on myoglobin and other proteins under macroscopic hydrated conditions report one fast relaxation process corresponding to free water dynamics which disappears upon dehydration, and slower relaxation processes associated with protein-water and protein dynamics still detected upon dehydration^43,45,58^. Schiro and co-authors have also shown using BDS that myoglobin confined in silica gels presents only slow relaxation processes as observed in our experiments, and little dynamic changes with the dehydration level of the sample^44^. This would tend to suggest that with the nanoconfinement, the dehydration impacts less the protein dynamics. The nanoconfinement could increase the packing of the toxin structure and thus stabilize the protein as observed for thermophilic proteins^47^. Repeating the experiments using different nanoconfinements and different dehydration conditions will be one way to test the role of both parameters in the temperature-resistance of the toxin.

Assessing nanoconfined protein dynamics is of interest as in cells geometrical nanoconfinement of proteins, for example in lipid microdomains (e.g. RAFT), regulate functional protein dynamics such as pore-formation, protein sorting and protein function^31,59,60^.

Protein dynamical structures detected under unusual experimental conditions (nanoconfinement and dehydration) show the robustness of protein structure and protein dynamics to a large range of conditions, going from crystal to dehydrated state in agreement with the sustainability of proteins in time and environmental conditions^61–63^. This is thanks to structural and dynamics diversity which also fits with the function of a protein provided by an ensemble of structures and dynamics rather than unique solutions^37,64^.

Importantly, the dielectric signal is protein conformation specific, evolves with the temperature of the thermal-treatments and is therefore sensitive to the perturbation of local atomic and molecular interactions. Thus the technique allows characterizing a protein in terms of conformation, stability (to temperature) and dynamics with multiple measures such as frequencies and maximum temperature positions. The technique could therefore be used to detect mutations by comparing the dielectric signals of a protein and its sequence-variants and diagnose the impact of mutations. Such experimental measurements would be useful to classify mutations and develop customized therapies accordingly.

Nanoconfinement allows us to investigate the dynamics of proteins at length scales comparable to the protein geometrical dimensions, which is expected to enhance the sensitivity of dielectric measurements and help detecting the impact of protein mutation. At the macroscopic scale, this ability is strongly limited by disorder-effects arising from a very broad distribution of possible protein conformations.

Presently, only the global dielectric signal can be used to distinguish molecular dynamics and conformations. It is impossible to determine the local dipoles responsible for the global signal or the scale of the collective fluctuations probed. In other words, whether the entire protein is probed or only sub-domains, and if so which one cannot be inferred using only the BDS data. This is because the sample is a mixture of three compounds, the protein, the PBS and protein bound-water. Each compound has many dipoles and the individual participation of each compound or how the individual signals combined to give the global signal is intractable.

**Figure 6:**
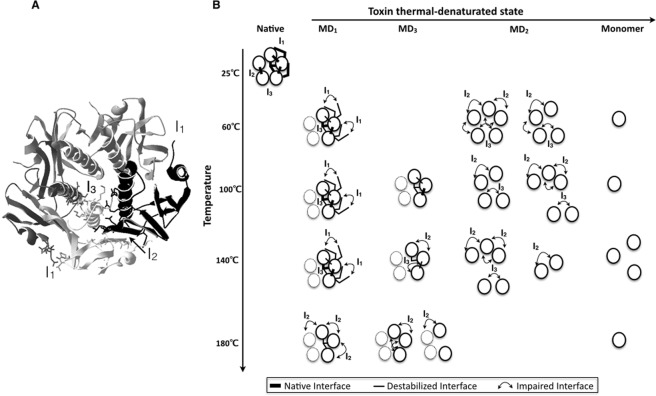
Model of the thermal denaturation of the cholera toxin B pentamer. (**A**) Interfaces in CtxB_5_. Ribbon representation of the atomic structure of CtxB_5_ with the three interface areas in sticks. Interface 1 (I_1_) where residues 92–93 on chain M interact with residues 1–4 on chain M − 1. Interface 2 (I_2_), the main toxin interface, where residues 23 to 31 on chain M interact with residues 96 to 103 on chain M + 1. Interface I_3_ (I_3_) where residues 63 to 74 of chains M − 1, M and M + 1 interact with one another. (**B**) The CtxB monomers are circles and the pentamer is composed of five circles, with two in dashed lines for the sake of clarity. The native interfaces and the monomers are not detected during the measurement. Only destabilized (fast motions) and impaired (slow motions) interfaces are detected. MD_1_ is associated with a pentamer having both fast and slow motions at all temperatures while MD_3_ pentamer has only fast motions at 100 °C, both at 140 °C and only slow at 180 °C. MD_2_ is associated with CtxB assembly intermediates that contain CtxB monomers with impaired interfaces and no interface.

What can be done instead is to challenge the data against known experimental and theoretical data on protein dynamics to draw hypotheses on the scale of the collective fluctuations detected by BDS. Putting the results into such perspective, we propose a model of the mechanism of the toxin thermal unfolding with the supplementary constraint that the model agrees with previously established mechanisms of the toxin unfolding and refolding^46,51,65,66^ (Fig. 6).

Amino acid side-chain motions determined by other experimental approaches and molecular dynamic simulations are several decades (≤nanoseconds) faster than the range of frequencies measured by BDS so it is reasonable to presume that such motions are not detected here.

According to molecular dynamics simulations and integrative approaches, microsecond to millisecond motions are attributed to loop, 2D and 3D structural elements whereas millisecond to second motions are folding and interdomain motions^1,3^. Microsecond to second dipole fluctuations on other proteins have been observed previously by BDS and attributed to protein backbone, large-scale domain or buried side chain motions^44,58^.

Because MD_1_ is the main peak detected from 60 °C to 140 °C, we will assume it corresponds to the most stable state of the toxin and its most folded average conformation, a ‘native-like’ pentamer. Because MD_3_ as MD_1_ has a narrow peak, and is similar in terms of frequency ranges but with slower relaxation times, we will assume it is a partially folded pentamer where the dipole have less compact environment allowing larger scale motions. Because MD_2_ is the slowest relaxation process and a broad peak, we will assume it to be the least folded CtxB species detected and a mixture of CtxB assembly intermediates (CtxB tetramers, trimers and dimers). Alternatively, MD_1_ could be a folded CtxB monomer, MD_3_ a slightly less folded monomer and MD_2_ multiple unfolded states of CtxB monomers. But because the three relaxation processes disappear at high temperatures, it is difficult to picture what would be the undetected species if unfolded monomers are detected. The first scheme is also consistent with the CtxB (dis)assembly and (un)folding intermediates previously identified macroscopically^46,65^. The activation energy of Proline trans-cis isomerization is 30 kcalmol^−1^, and maybe the toxin relaxation processes and dipole fluctuations are under the influence of such reaction. As the toxin assembly is inhibited by the isomerization of Pro93 which slows down the formation of the main β-interface, it is possible although speculative^46^.

Under the first scheme, it is reasonable to assume that the toxin subunit interfaces are monitored during an experiment since CtxB assembly intermediates are detected (MD_2_). It follows that folded and unfolded CtxB monomers would be among the undetected species. The native toxin pentamer has three distinct interface areas I_1_, I_2_ and I_3_ as shown in Fig. 6A, but native interfaces are unlikely to be detected because they involve local atomic motions with fluctuations over the nanosecond scale^3^. In MD_1_, fast and slow relaxation times are detected and as the toxin unfolds with high temperature treatments only slow relaxation times are detected for MD_2_ and MD_3_. The toxin interfaces I_1_, I_2_ and I_3_ would exist as native (nanosecond scale not detected), destabilized (microsecond to millisecond fluctuations: 2D/3D segment mobility) and impaired with larger and slower motions (millisecond to second: domain motion) (Fig. 6B). These assumptions are consistent with the microsecond to millisecond time-scale motions, measured by hydrogen exchange, involved in protein thermal unfolding^67^. The model assumes that CtxB intermediates are detected through the slow motion interfaces, the interfaces with no adjacent chains are assumed undetected. Having I_1_, I_2_ and I_3_ interfaces with slow motions in a monomer, is assumed to trigger the monomer dissociation extremely fast.

Now, in MD_1_ even at 180 °C fast relaxation times (microsecond to millisecond) are observed but for different maximum temperature positions than at 140 °C. This suggests that some undetected native interfaces present in MD1 at 140 °C, become destabilized and detected at 180 °C but at different maximum positions than the one detected at 140 °C because they are different interfaces (Fig. 6). Alternatively, all the interfaces in MD_1_ could be destabilized and detected with fast motions from 60 °C, and the change in maximum temperature positions with the thermal treatements could indicate local environmental changes around the dipoles with fast relaxation times due to local unfolding with no consequences at larger scales. This would have to be further investigated.

The model highlights the large combinatory of interface dynamic states within a single monomer, the CtxB assembly intermediates and the pentamers. The dielectric loss measurement is performed on hundreds of nanopores, each containing some toxin molecules so it is capturing a large statistics of structural states, referred to as average conformations and appearing within each molecular dynamics.

## Conclusion

The molecular dynamics of distinct protein conformations, among which conformations rarely detected macroscopically, become accessible experimentally by combining nanoconfinement with broadband dielectric spectroscopy. This novel biophysical approach captures the dipole fluctuations underlying the molecular dynamics of average protein conformations whose slow motions associate with protein unfolding. The level of description provided by the BDS offers a large experimental potential to study protein dynamics and is an experimental equivalent to molecular dynamics simulation over the frequency range from 10^3^ Hz to 10^6^ Hz.

## Supplementary information

Supplementary information
