# Supplementary material for: Experimental Protein Molecular Dynamics: Broadband Dielectric Spectroscopy coupled with nanoconfinement
Source: Sci Rep. 2019 Nov 29;9:17988. doi: 10.1038/s41598-019-54562-8 (PMC6884508; doi:10.1038/s41598-019-54562-8)
Supplement: Supplementary file 1 — Supplementary information [file 41598_2019_54562_MOESM1_ESM.pdf]

Supplementary Information

Experimental Protein Molecular Dynamics: Broadband Dielectric Spectroscopy coupled with nanoconfinement

Laëtitia Bourgeat[1,2], Anatoli Serghei[2*] and Claire Lesieur[1,3*]

[1]AMPERE, CNRS, Univ. Lyon, 69622, Lyon, France

[2]IMP, CNRS, Univ. Lyon, 69622, Lyon, France

[3]Institut Rhônalpin des systèmes complexes, IXXI-ENS-Lyon, 69007, Lyon, France

*Corresponding authors: claire.lesieur@ens-lyon.fr; anatoli.serghei@univ-lyon1.fr

Supplementary figures

Supplementary Figure S1

Supplementary Figure S2

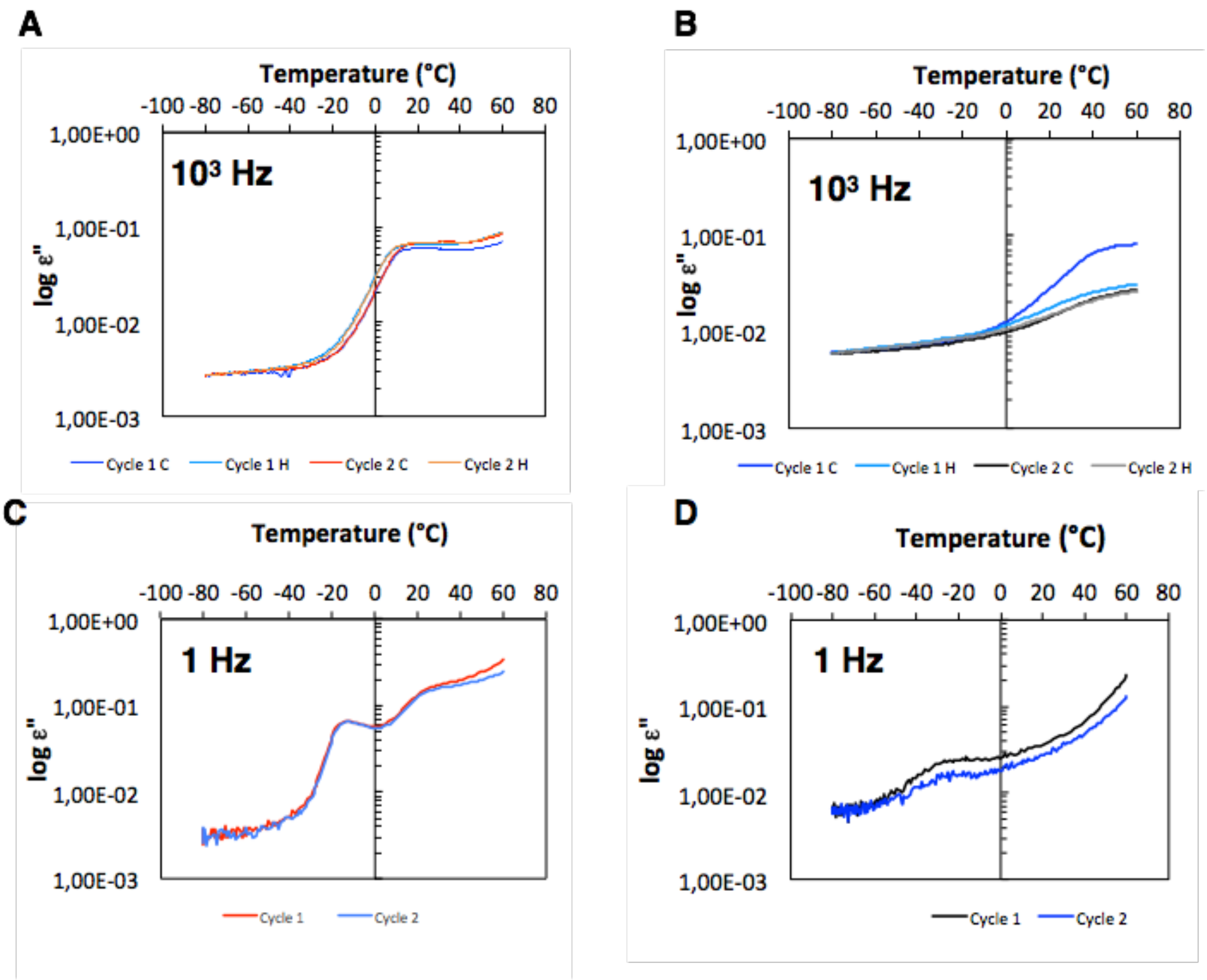

Supplementary Figure S1. Samples stability. A. Toxin stability at 60°C at 10^3Hz. The toxin sample is heated for three hours at 60°C and the dielectric loss is measured from 60°C to -80°C (Cycle 1 C, C for cooling). The dielectric loss is then measured from -80°C to 60°C (Cycle 1 H, H for heating). The toxin sample is heated again for three hours at 60°C and the cooling/heating measurement repeated (Cycle 2C and cycle 2H). **B.** PBS buffer control stability at 60°C at 10^3Hz. The PBS buffer control sample undergoes same treatment as the toxin sample. **C.** Toxin stability at 60°C at 1 Hz. Legend as in **A. D.** PBS buffer control stability at 60°C at 1 Hz. Legend as in **B.**

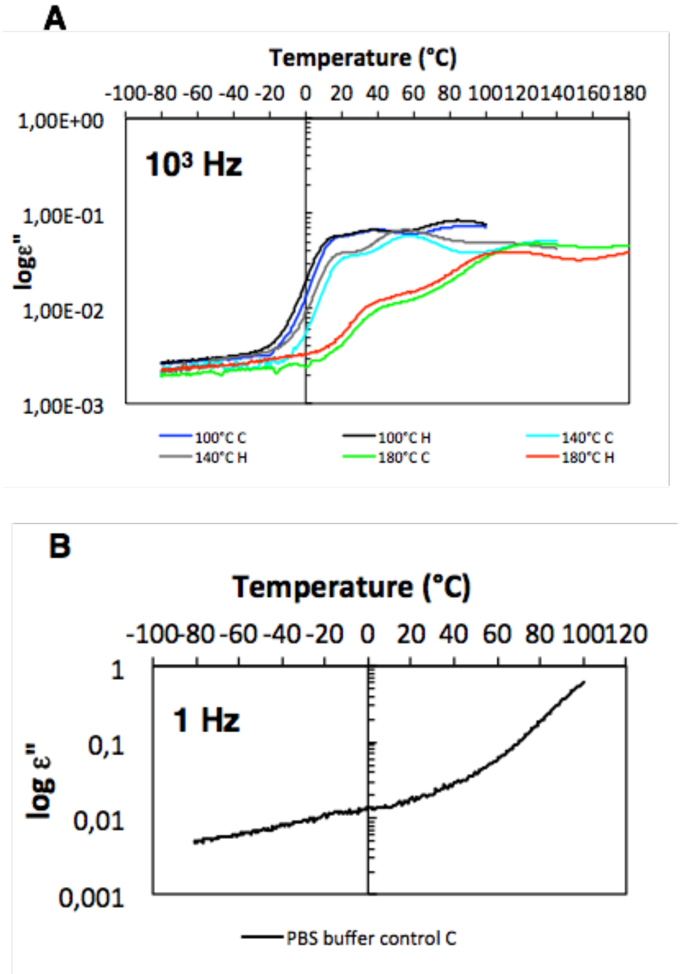

Supplementary Figure S2. Sample signals after thermal treatments higher than 60°C. A. Signal hysteresis. The signals of the toxin samples for the cooling (C) and heating (H) cycles after thermal treatments at 100°C, 140°C and 180°C and measurement at 10^3 Hz. **B.** PBS buffer control stability at 100°C. The cooling signal of the PBS buffer control sample is shown after three hours at 100°C.
